# Awareness and uptake of layered HIV prevention programming for young women: analysis of population-based surveys in three DREAMS settings in Kenya and South Africa

**DOI:** 10.1186/s12889-019-7766-1

**Published:** 2019-10-30

**Authors:** Annabelle Gourlay, Isolde Birdthistle, Nondumiso Thandiwe Mthiyane, Benedict O. Orindi, Sheru Muuo, Daniel Kwaro, Maryam Shahmanesh, Kathy Baisley, Abdhalah Ziraba, Sian Floyd

**Affiliations:** 1 Faculty of Epidemiology and Population Health, London School of Hygiene & Tropical Medicine, Keppel Street, London, WC1E 7HT UK; 2 Africa Health Research Institute, Durban, KwaZulu-Natal South Africa; 3 Africa Population and Health Research Center, Nairobi, Kenya; 4 Centre for Global Health Research, Kenya Medical Research Institute, Kisumu, Kenya; 5 Institute for Global Health, University College London, London, UK

**Keywords:** HIV prevention, Adolescent girls, Implementation, Evaluation, Complex intervention

## Abstract

**Background:**

The DREAMS Partnership is an ambitious effort to deliver combinations of biomedical, behavioural and structural interventions to reduce HIV incidence among adolescent girls and young women (AGYW). To inform multi-sectoral programming at scale, across diverse settings in Kenya and South Africa, we identified who the programme is reaching, with which interventions and in what combinations.

**Methods:**

Randomly-selected cohorts of 606 AGYW aged 10–14 years and 1081 aged 15–22 years in Nairobi and 2184 AGYW aged 13–22 years in uMkhanyakude, KwaZulu-Natal, were enrolled in 2017, after ~ 1 year of DREAMS implementation. In Gem, western Kenya, population-wide cross-sectional survey data were collected during roll-out in 2016 (*n* = 1365 AGYW 15–22 years). We summarised awareness and invitation to participate in DREAMS, uptake of interventions categorised by the DREAMS core package, and uptake of a subset of ‘primary’ interventions. We stratified by age-group and setting, and compared across AGYW characteristics.

**Results:**

Awareness of DREAMS was higher among younger women (Nairobi: 89%v78%, aged 15-17v18–22 years; uMkhanyakude: 56%v31%, aged 13-17v18–22; and Gem: 28%v25%, aged 15-17v18–22, respectively).

HIV testing was the most accessed intervention in Nairobi and Gem (77% and 85%, respectively), and school-based HIV prevention in uMkhanyakude (60%). Among those invited, participation in social asset building was > 50%; > 60% accessed ≥2 core package categories, but few accessed all primary interventions intended for their age-group. Parenting programmes and community mobilisation, including those intended for male partners, were accessed infrequently.

In Nairobi and uMkhanyakude, AGYW were more likely to be invited to participate and accessed more categories if they were: aged < 18 years, in school and experienced socio-economic vulnerabilities. Those who had had sex, or a pregnancy, were less likely to be invited to participate but accessed more categories.

**Conclusions:**

In representative population-based samples, awareness and uptake of DREAMS were high after 1 year of implementation. Evidence of ‘layering’ (receiving multiple interventions from the DREAMS core package), particularly among more socio-economically vulnerable AGYW, indicate that intervention packages can be implemented at scale, for intended recipients, in real-world contexts. Challenges remain for higher coverage and greater ‘layering’, including among older, out-of-school AGYW, and community-based programmes for families and men.

## Background

Adolescent girls and young women (AGYW) aged 15–24 years remain at high risk for HIV infection compared to their male counterparts, particularly in sub-Saharan Africa [1, 2]. The estimated 450,000 new HIV infections among AGYW globally in 2015 [1] is far from the UNAIDS goal to reduce annual new infections to below 100,000 by 2020 [1].

The DREAMS (Determined, Resilient, Empowered, AIDS-free, Mentored, and Safe lives) Partnership is an ambitious public-private investment, established in 2015 to reduce the rate of new HIV infections among AGYW in ten sub-Saharan African countries [3, 4]. DREAMS is based on the principle that ‘combination HIV prevention’ [5]– an approach to reduce HIV transmission through integrated behavioural, biological and structural interventions tailored to the needs of a population – is essential. In the case of DREAMS, the multiple sources of HIV risk for adolescent girls and young women are conceptualised through a theory of change model and are to be addressed through a package of ‘layered’ evidence-based interventions [6]. ‘Layering’ is defined by the President’s Emergency Fund for AIDS Relief (PEPFAR) as “at the individual level…to provide multiple interventions or services from the DREAMS core package to each DREAMS recipient (i.e. AGYW)” while “layering also takes into account contextual level interventions (i.e. those that are not delivered directly to an AGYW but from which she may benefit)” [7].

**Fig. 1:**
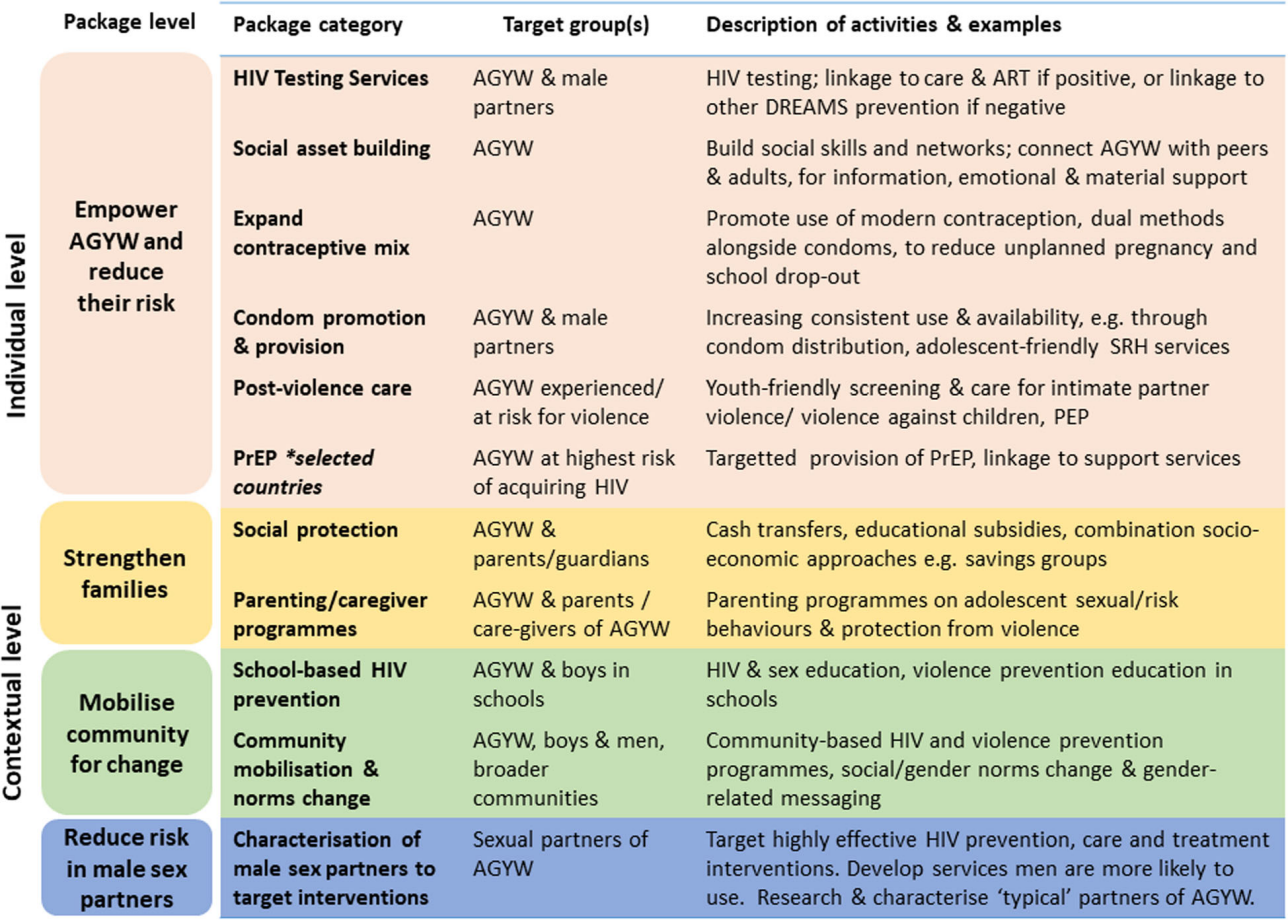
Framework for DREAMS core package of interventions

Figure 1 illustrates the key components of the DREAMS core package of interventions, grouped into categories (e.g. social protection), which in turn are organised by levels (e.g. ‘strengthen families’, which can also be described as ‘contextual level’) [6, 8, 9]. At the individual-level, interventions aim to empower AGYW and reduce their risk of HIV and violence, for example, through access to HIV testing and youth-friendly sexual and reproductive health services, or social asset building interventions such as ‘safe spaces’ where AGYW can meet with mentors and peers for social support, courses, and links to services. Contextual-level interventions are intended to strengthen families, for example, economically and through parenting support, and to mobilise communities more broadly to address social norms, including through schools. The core package also includes strategies to reduce an AGYW’s risk of acquiring HIV from a male partner, through the expansion of essential HIV and/or prevention services including HIV testing, linkage to ART and voluntary medical male circumcision (VMMC). Each country has subsequently selected a minimum package of ‘primary’ interventions from the core package, some that are intended for all AGYW and some that are for particular age groups. ‘Secondary’ interventions are based on need, rather than being intended for all AGYW, for example, post-violence care services for those who have experienced violence [7].

Complex interventions are proliferating, for example, with the ‘Sauti’ initiative in Tanzania, ‘She Conquers’ in South Africa, and ‘DREAMS-like’ AGYW programs funded by the Global Fund for AIDS, TB and Malaria [10–12]. Investments in packages of services are also promoted to address the broader needs of adolescents, with global calls to go beyond school-based education to involve families, communities and media in adolescent development [13, 14]. To date, however, there is sparse empirical evidence that such complex adolescent interventions can be taken to scale (at district level, for example) and implemented effectively in real-world, non-trial conditions. Their effectiveness will depend on the intensity and quality with which they are delivered and whether they are accessed by young people and related target populations.

The question of how to achieve effective delivery and reach is fundamental to addressing the ‘implementation gap’ [15, 16]. It has been argued that it is now imperative to fill the gap between what we know works and what can be achieved in reality. Fauci and colleagues emphasise there should be “no more excuses” and that “we have the tools to end the HIV/AIDS pandemic”, although “from a practical standpoint, this will be difficult and will require aggressive implementation of the biomedical research advances that have been made…” [15].

As part of an independent impact evaluation of DREAMS among representative samples of AGYW in Kenya and South Africa [17], we investigate the population-level uptake of DREAMS, specifically the awareness and uptake of any and multiple (‘layered’) DREAMS interventions after the first year of implementation.

## Methods

### Evaluation settings

The impact evaluations are underway in three diverse settings: urban, informal settlements in Nairobi, Kenya; Gem constituency in rural Siaya county, western Kenya; and uMkhanyakude in rural KwaZulu-Natal, South Africa. Descriptions of each setting and methods for the evaluation protocol have been described previously [17]. HIV prevalence and incidence are historically high in these settings [18–22].

### Implementation of DREAMS interventions

In all three settings, DREAMS interventions were first introduced in 2016: January–February in Nairobi; April in Gem; and May in uMkhanyakude. Implementation of services was staggered and took time to scale-up, especially for newer services without pre-existing infrastructure, such as social asset building. All services apart from Pre-Exposure Prophylaxis (PrEP) were being provided by March 2016 in Nairobi, by late 2016 in uMkhanyakude, and by January 2017 in Gem. Details about the timing and model of DREAMS delivery in each site are summarised elsewhere [23].

DREAMS implementers sought to reach and invite the most vulnerable AGYW to participate, although the way this was operationalised differed by setting [24]. All AGYW were eligible for DREAMS interventions in uMkhanyakude, the area having been identified as high priority after an extensive geographic mapping exercise. In both sites in Kenya, AGYW at highest risk were targeted for and invited to participate in DREAMS interventions. Examples of targeting included to invite those who had children/were pregnant, were school-age and out of school, or were sexually abused, through door-to-door home visits followed by enrolment interviews. They were identified through the ‘Girl Roster’ census method, supplemented by local experience of community-based organisations [25].

### Evaluation study design and data collection

The impact evaluation design [17] leverages long-standing demographic and HIV survey platforms in each setting: the Nairobi Urban Health and Demographic Surveillance System (NUHDSS); the Kenya Medical Research Institute/CDC site in western Kenya; and the Africa Health Research Institute platform in KwaZulu-Natal, South Africa [26–28]. In brief, the design includes three main components: 1) general population (cross-sectional) surveys of women and men, to be repeated over 2–3 time points; 2) nested within this, randomly-selected cohorts of AGYW stratified on age, for more detailed understanding of trajectories and transitions over time, and with annual follow-up for 2 years; and 3) process evaluation activities to document the implementation and adaptation of DREAMS in each context.

The first time period for data collection for the impact evaluation was during 2016–2018. In Nairobi, it was from March–July 2017, after one full year of DREAMS implementation. All men and women aged 15–49 years were eligible for the general population survey, and AGYW aged 10–14, 15–17 and 18–22 years were enrolled into nested cohorts. Adolescents aged 10–14 were included given DREAMS also intends to deliver prevention interventions to this age group, and because it could make a difference to reach AGYW relatively early [6]. In uMkhanyakude, data were collected during January–December 2017 for the general population survey (residents aged ≥15 years), while nested cohorts of AGYW aged 13–17 years and 18–22 years were enrolled between May 2017 and February 2018. In Gem, a population-wide bio-behavioural survey was conducted during early roll-out of DREAMS, from May to September 2016, for which ~ 25% of randomly-selected households in the demographic platform were eligible, including all their household members.

In Nairobi and uMkhanyakude, DREAMS-specific questions were embedded in the population-wide survey platforms, covering overall awareness of and self-reported invitation to participate in DREAMS, as well as awareness and recent (prior 12 months) usage of individual DREAMS interventions. Those who had participated in an intervention in the previous 12 months were asked if they identified the activity as a DREAMS service, and which organisation delivered it. In Gem, a relatively limited set of data were collected that included awareness of and usage (ever) of individual interventions. Socio-demographic data were collected in all settings. These general-population survey data were used for analyses among women aged ≥25 years and men, and (for Gem only) for AGYW analyses.

As well as the questions included in the general population surveys, additional questions were asked in the nested cohort interviews, and so cohort data were used for analyses among AGYW in Nairobi and uMkhanyakude. For girls aged 10–14 years in Nairobi, the questions were modified and so data from this age group were analysed separately.

### Analysis

Descriptive analyses were conducted using *Stata15* to summarise proportions of respondents who reported awareness of, self-reported invitation to participate in, and uptake of DREAMS interventions, categorised using the DREAMS core package framework (Fig. 1) and by primary interventions (Additional file 1). We defined uptake as any usage in the last 12 months (Nairobi and uMkhanyakude), or ever (Gem), regardless of whether the respondent specifically recalled the intervention was delivered through DREAMS. For some curriculum-based interventions, e.g., those to be delivered in ‘safe spaces’ for AGYW, this implies participation in at least one session, rather than completion of all sessions.

We analysed usage of individual intervention categories from the core package, for example the proportion who used HIV testing services or social protection interventions (Fig. 1). To assess evidence of ‘layering’ we:

- summarised proportions who had received interventions from multiple core package categories (≥2 or ≥ 3);
- determined combined usage of intervention categories from across different *levels*, for example, empowering AGYW (individual-level) plus interventions that aim to strengthen families and/or mobilise communities (contextual-level); and
- examined the number of primary intervention categories used and the proportion receiving the complete ‘package’ of intended primary interventions (Additional file 1) [17].

Analyses were stratified by sex, age-group, setting, and invitation to participate in DREAMS. Individual- and contextual-level interventions were summarised for AGYW; contextual-level for older women and men (as the DREAMS core package also aims to reach families, male partners and communities through contextual-level interventions) (Fig. 1). We restricted analyses among males to those aged 15–34 years, to reflect the typical age range of partners of AGYW in these settings [29]. We also made comparisons across AGYW characteristics. Selection of characteristics for analyses was informed by the programme implementation and targeting of AGYW for DREAMS interventions. Variable categories were standardised across settings where feasible, to aid comparisons.

To understand who is reached by DREAMS, univariable analyses were done first and then used to guide multivariable logistic regression. These analyses were conducted with AGYW cohort data (Nairobi and uMkhanyakude only), to quantify associations between AGYW characteristics and measures of DREAMS uptake, specifically: (i) invitation to participate in DREAMS, and (ii) uptake of multiple (‘layered’) core package intervention categories. Variables were added in a forward step-wise fashion, and retained in the model if there was statistical evidence of association with the outcome (*p* < 0.10), based on likelihood ratio tests.

### Reporting

The STROBE reporting guidelines were used to guide synthesis and standardise reporting of our results across settings (Additional file 2) [30].

### Ethics

Ethics approval was received by research ethics committees at the London School of Hygiene and Tropical Medicine (Ref 11835) and within the host countries: the Biomedical Research Ethics Committee of the University of KwaZulu-Natal, South Africa; the African Medical and Research Foundation Health Africa for the research in Nairobi, Kenya; and the Kenyan Medical Research Institute for the research in Siaya, Kenya. Written informed consent was obtained from all participants, in addition to assent from legal minors with guardian consent (for those aged < 18 years). Compensation for participation in the research included refreshments, soap and/or reimbursement for transport costs, where applicable.

## Results

### Participant numbers and characteristics

Overall, 606 AGYW aged 10–14 years, 547 aged 15–17 years and 534 aged 18–22 years were recruited into nested cohorts in Nairobi (response rate of 61% for AGYW aged 15–22 years, *n* = 1770 eligible); 1148 aged 13–17 years and 1036 aged 18–22 years in uMkhanyakude (response rate of 85% for all AGYW aged 13–22 years, *n* = 2555 eligible) (Tables 1 and 2). In Gem, 481 AGYW aged 15–17 years and 884 aged 18–22 years participated in the general population survey and answered questions on DREAMS.

**Table 1:** Profiles of AGYW aged 13/15–22 in Nairobi, uMkhanyakude (nested cohorts) and Gem (general population survey)

Characteristics of AGYW	Nairobi, Kenya				uMkhanyakude, South Africa				Gem, Kenya			
	15–17		18–22		13–17		18–22		15–17		18–22	
	Total		Total		Total		Total		Total		Total	
	*n* = 547	%	*n* = 534	%	*n* = 1148	%	*n* = 1036	%	*n* = 481	%	*n* = 886	%
DREAMS awareness												
Heard of DREAMS	489	89.4	414	77.5	627	54.6	324	31.3	135	28.1	223	25.2
Not heard of DREAMS	58	10.6	120	22.5	521	45.4	710	68.7	346	71.9	661	74.8
Informal settlement site Nairobi												
Korogocho	317	58.0	300	56.2								
Viwandani	230	42.0	234	43.8								
Residence area												
Rural					727	63.9	661	64.4				
Peri-urban					351	30.8	309	30.1				
Urban					60	5.3	57	5.6				
Marital status												
Never	534	97.6	309	57.9	1148	100	1035	99.9	454	94.6	544	61.6
Previously married/cohabiting	1	0.2	32	6.0	0	0	0	0	0	0	15	1.7
Currently married/cohabiting	12	2.2	193	36.1	0	0	1	0.1	26	5.4	324	36.7
Education^a^												
None	0	0	7	1.3	0	0	2	0.2	1	0.2	5	0.6
Currently in school	459	83.9	167	31.3	1128	98.3	516	49.9	412	85.7	11	1.3
Not in school, some primary	57	10.4	126	23.6	5	0.4	27	2.6	57	11.9	443	50.3
Not in school, some secondary	29	5.3	210	39.3	15	1.3	433	41.9	11	2.3	401	45.6
Not in school, some tertiary	2	0.4	24	4.5	0	0	56	5.4	0	0	20	2.3
Recent/current employment^b^												
No	527	96.3	421	78.8	1129	98.6	990	96.1	469	98.5	646	74.0
Yes	20	3.7	113	21.2	16	1.4	40	3.8	7	1.5	227	26.0
Self-assessed household poverty												
Very poor	66	12.1	73	13.7								
Moderately poor	435	79.5	423	79.2								
Not poor	46	8.4	38	7.1								
Received government social grant												
No					338	30.4	775	75.0				
Yes (child-care/foster-child)					773	69.6	259	25.0				
Socio-economic status												
Low					380	34.2	347	36.0				
Middle					386	34.8	361	37.4				
High					344	31.0	256	26.6				
Food insecure^c^												
No	351	65.5	366	68.9	898	78.2	603	58.3				
Yes	185	34.5	165	31.1	250	21.8	432	41.7				
Number of household assets												
0to5	115	21	108	20.2								
6to7	167	30.5	190	35.6								
8to9	160	29.3	152	28.5								
10to15	105	19.2	84	15.7								
Number of individual assets												
0to3	48	8.8	40	7.5								
4to6	331	60.5	370	69.3								
7to10	168	30.7	124	23.2								
Ever had sex												
No	479	87.9	163	30.5	999	87.9	279	27.8	373	77.6	221	25.0
Yes	66	12.1	371	69.5	137	12.1	724	72.2	108	22.5	663	75.0
Ever pregnant												
No	514	94.0	266	49.8	1077	94.5	499	50.3	431	90.2	413	47.2
Yes	31	5.7	268	50.2	63	5.5	494	49.7	47	9.8	462	52.8
Ever given birth												
No	519	94.9	286	53.6	1099	96.3	548	53.9				
Yes	26	4.8	248	46.4	42	3.7	469	46.1				
HIV status (self-reported)												
Positive	15	2.7	7	1.3	27	2.4	108	10.4	9	1.9	28	3.2
Negative	422	77.1	467	87.5	183	15.9	313	30.2	331	68.8	815	92.2
Unwilling to share	22	4.0	22	4.1								
Never tested/unknown	88	16.1	38	7.1	938	81.7	615	59.4	141	29.3	41	4.6

^a^ ‘Some primary’ indicates completion of at least some primary education; Gem: question on current schooling only asked to a subset of adolescents aged 13–17 years

^b^ Nairobi: Yes = recently employed within the last month; uMkhanyakude: Yes = currently employed; Gem: Yes = has a defined occupation or ‘other’ occupation with source of income from a job or business, other than student, housewife, unemployed, or other

^c^ Nairobi: Girl or household member went to sleep at night hungry because there was not enough food in past 4 weeks; uMkhanyakude: Girl or household member ever skipped or cut the size of a meal because there was not enough money for food

**Table 2:** Profile, invitation to participate, and uptake of DREAMS core package among AGYW aged 10–14, Nairobi

Characteristics of AGYW	Cohort profile		Invited to participate		Number of core package categories accessed							
	Total		n	% (row)	0		1		2		3+	
	N	% (col)			n	%	n	%	n	%	n	%
Total	606		290	47.9	141	23.3	124	20.5	108	17.8	233	38.5
Age												
10–12	372	61.4	163	43.8	101	27.2	83	22.3	66	17.7	122	32.8
13–14	234	38.6	127	54.3	40	17.1	41	17.5	42	17.9	111	47.4
Informal settlement site Nairobi												
Korogocho	323	53.3	192	59.4	39	12.1	61	18.9	68	21.1	155	48
Viwandani	283	46.7	98	34.6	102	36	63	22.3	40	14.1	78	27.6
Currently enrolled in school												
No	5	0.8	1	20.0	2	40	2	40	0	0	1	20
Yes	601	99.2	289	48.1	139	23.1	122	20.3	108	18	232	38.6
Current schooling and school progress												
Not in school	5	0.8	1	20.0	2	40	2	40	0	0	1	20
2+ classes behind at school^a^	177	29.2	99	55.9	39	22	38	21.5	28	15.8	72	40.7
< 2 classes behind at school^a^	424	70.0	190	44.8	100	23.6	84	19.8	80	18.9	160	37.7
Paid jobs/activities, last 6 months												
No	577	95.2	275	47.7	136	23.6	122	21.1	102	17.7	217	37.6
Yes	29	4.8	15	51.7	5	17.2	2	6.9	6	20.7	16	55.2
Family food insecurity^b^												
Never	227	37.5	95	41.9	64	28.2	47	20.7	38	16.7	78	34.4
Sometimes	331	54.6	165	49.8	70	21.1	67	20.2	65	19.6	129	39
Often	47	7.8	29	61.7	7	14.9	9	19.1	5	10.6	26	55.3
Number of people sleep in same room												
0–1	84	13.9	35	41.7	22	26.2	17	20.2	12	14.3	33	39.3
2–3	239	39.4	108	45.2	63	26.4	43	18	44	18.4	89	37.2
4+	283	46.7	147	51.9	56	19.8	64	22.6	52	18.4	111	39.2
Romantic relationships												
Never	541	89.4	263	48.6	128	23.7	109	20.1	94	17.4	210	38.8
Previously	41	6.8	18	43.9	7	17.1	7	17.1	10	24.4	17	41.5
Currently in relationship (not married)	23	3.8	8	34.8	6	26.1	8	34.8	3	13	6	26.1
Ever had sex												
No	593	97.9	285	48.1	137	23.1	123	20.7	106	17.9	227	38.3
Yes	12	2.0	5	41.7	3	25	1	8.3	2	16.7	6	50
Sexually exploited^c^												
No	566	93.4	275	48.6	131	23.1	118	20.8	100	17.7	217	38.3
Yes	40	6.6	15	37.5	10	25	6	15	8	20	16	40
Physical violence, last 6 months												
No	507	83.7	244	48.1	120	23.7	98	19.3	89	17.6	200	39.4
Yes (slapped, hit, physically hurt)	99	16.3	46	46.5	21	21.2	26	26.3	19	19.2	33	33.3
Verbal violence, last 6 months												
No	407	67.2	198	48.6	93	22.9	90	22.1	75	18.4	149	36.6
Yes (teased, bullied or threatened)	199	32.8	92	46.2	48	24.1	34	17.1	33	16.6	84	42.2

^a^ includes enrolled in school but school holiday/ vacation from school currently; ^b^ ever been a time when your family did not have enough food because they had no money; ^c^reported being threatened, coerced or being forced into being touched or having (first) sex, or said they were unwilling to have (first) sex, or they were ever forced into/attempted sex by an adult (childhood experiences), or reported being touched in the last 6 months in a way they did not want to be touched

Most AGYW respondents were never married, or in the case of girls aged 10–14 years in Nairobi, had never had romantic relationships. Most aged < 18 years were in school, while the majority aged 18–22 years in Nairobi were out of school and had completed at least some primary or secondary education, compared to similar proportions in and out of school among AGYW aged 18–22 years in uMkhanyakude. Very few AGYW aged 18–22 years were currently employed either part-time or full-time in uMkhanyakude (~ 4%), in contrast to 21% and 26% of those aged 18–22 years in Nairobi and Gem, respectively. Proportions who had ever had sex were similar in Nairobi and uMkhanyakude, and rose by age group, from 2% of girls aged 10–14 years in Nairobi, to 12% of those aged 15–17 years and 13–17 years respectively in Nairobi and uMkhanyakude, and ~ 70% among those aged 18–22 years in both settings. In Gem, a higher proportion of AGYW aged 15–17 years reported having had sex (22%); 75% among AGYW aged 18–22 years. Around half of those aged 18–22 years in each setting had been pregnant. Few respondents self-reported HIV-positive (2% of those who had ever tested in Nairobi; 3% for Gem; 6% for uMkhanyakude).

The majority of men aged 15–34 years were never married, ranging from 53% in Nairobi, to 69% in Gem and 99.5% in uMkhanyakude (Additional file 3). Higher proportions were employed in Nairobi compared to Gem, with low levels of employment in uMkhanyakude. A greater proportion of men aged 15–34 years were in school in uMkhanyakude compared to Kenya, at least in part reflecting the younger age distribution in this setting.

### Awareness of DREAMS

After 1 year of implementation, AGYW awareness of DREAMS was higher in Nairobi (80% aged 10–14 years, data not shown, 89% aged 15–17 years, 78% aged 18–22 years) than uMkhanyakude (55% aged 13–17 years, 31% aged 18–22 years). During the initial 6 months of roll-out in Gem, about one-quarter of AGYW were aware of DREAMS (Table 1), with the proportion increasing each month (data not shown). Lower proportions of men (Nairobi: 39% and 34%; Gem: 13% and 11%, for ages 15–34 years and 35–49 years respectively) and women aged 25–49 years (Nairobi: 64%; Gem: 20%) had heard of DREAMS (Additional file 3).

The primary sources of information about DREAMS in Nairobi were word of mouth and community-based/ non-governmental organisations for AGYW (Additional file 4), as for men and older women (Additional file 5). School was the key information source for AGYW in uMkhanyakude, and commonly cited among school-aged girls and boys in Nairobi.

Awareness of specific DREAMS interventions among AGYW was generally high, more so for individual-level interventions than contextual-level. For most interventions, the majority of AGYW reporting participation within the last 12 months also recognised the intervention as being delivered through DREAMS, and recognition of DREAMS was generally higher among those aged 13/15–17 years than those aged 18–22 years (Additional files 6 and 7, example shown for Nairobi).

### Uptake of individual intervention categories of the DREAMS core package

**Fig. 2:**
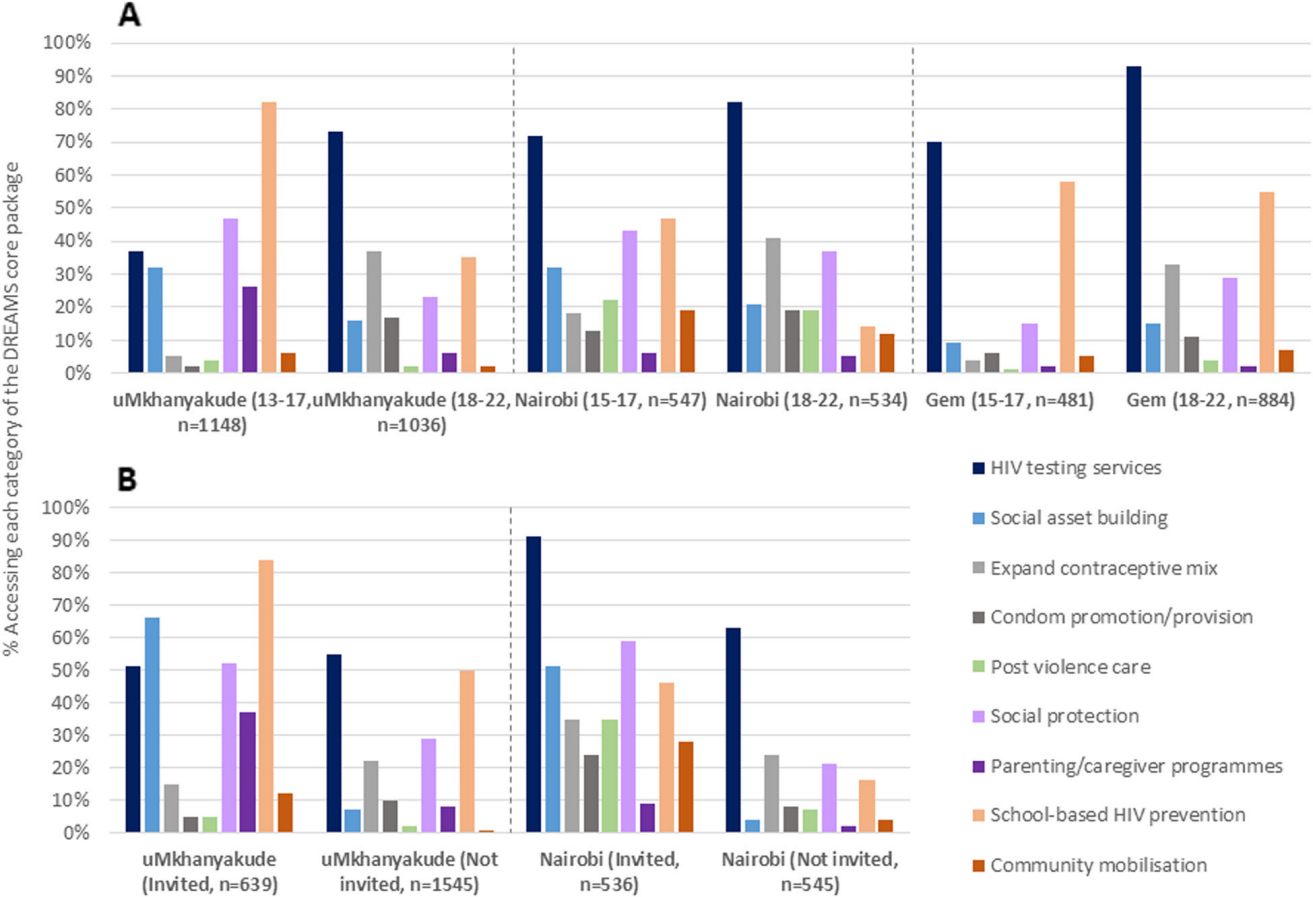
Uptake* of DREAMS core package** in three settings by: **a** age; **b** DREAMS invitation. Footnote:*uMkhanyakude and Nairobi: Participated *in the last 12 months* (datasets from 2017); Gem: *ever* participated (dataset from 2016); Uptake regardless whether or not the intervention was identified as a ‘DREAMS programme’ **Interventions aligned with PEPFAR Core Package outlined to countries in 2015

HIV testing was the most accessed intervention category among AGYW in Kenyan settings (77% overall in Nairobi and 85% in Gem), while in uMkhanyakude, school-based HIV and violence prevention was most accessed overall (60% among all AGYW aged 13–22 years; 80% among those aged 13–22 years and in school) and among girls aged 13–17 years (Fig. 2, panel A). In all three settings, expanding the contraceptive method mix and condom promotion/provision were more frequently used by AGYW aged 18–22 years than younger AGYW, while in uMkhanyakude and Nairobi, social asset building and social protection were more commonly accessed by younger AGYW aged < 18 years than those aged 18–22 years.

Among AGYW invited into DREAMS in Nairobi and uMkhanyakude, almost all participated in (any) DREAMS interventions (≥97% in both settings, data not shown), and recent participation in most intervention categories was substantially higher compared to those not invited (Fig. 2, panel B). Differences were greatest for social asset building and social protection interventions, usage rising to > 50% of those invited (versus < 10% among those not invited). Participation in post-violence care, community mobilisation/norms change, and parenting/caregiver interventions was also markedly higher among those invited compared to those not invited. However, parenting and community-based programmes were accessed infrequently overall, in all settings.

**Fig. 3:**
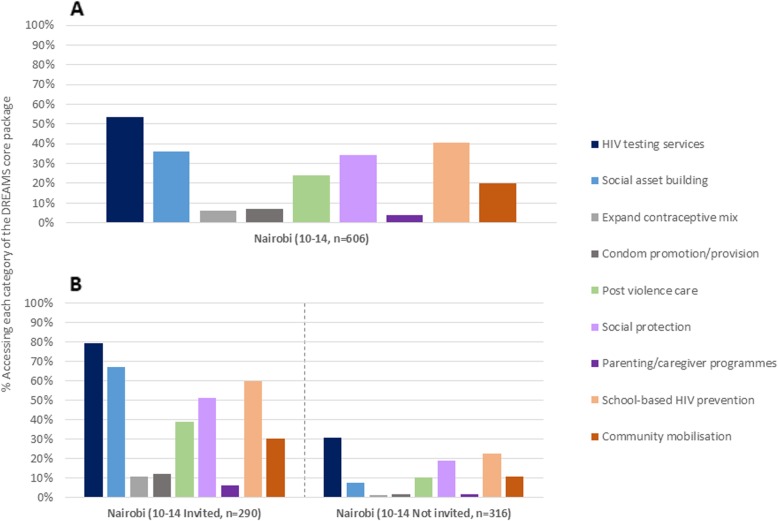
Uptake* of DREAMS core package** in Nairobi, 10–14 year-olds: **a** overall, **b** by DREAMS invitation. Footnote: *Participated *in the last 12 months* (dataset from 2017); Uptake regardless whether or not the intervention was identified as a ‘DREAMS programme’ **Interventions aligned with PEPFAR Core Package outlined to countries in 2015

Patterns of uptake among 10–14 year-olds in Nairobi were broadly similar to those among 15–17 s, with HIV testing services, school-based prevention, social asset building and social protection the most used intervention categories, although levels of HIV testing were lower (Fig. 3). However, among 10–14 s invited to participate in DREAMS, recent usage of HIV testing services rose to 80%, with substantial differences in participation between those invited versus not invited for all intervention categories.

In Nairobi and Gem, usage of relevant DREAMS services, for example community mobilisation, cash transfers (social protection) and parenting/caregiver programmes, was generally low among women aged 25–49 years (≤11% for each intervention in both settings) and men aged 15–49 years (≤5%, Nairobi and Gem) (Additional file 8). The exceptions were HIV testing services, accessed by 54% of men in Nairobi in the last 12 months (55% among men aged 15–34 years), and school-based HIV education, accessed by 31% of males aged 15–34 years who were in school (7% among all men aged 15–34 years). Few men had recently accessed VMMC in this setting (2% overall and 2% among men aged 15–34 years). In Gem, 89% of men had ever participated in HIV testing services (88% among those aged 15–34 years), 3% (*n* = 2828) were circumcised in a health facility in 2016 (4% of men aged 15–34 years, *n* = 2142), and 43% of men aged 15–34 years had ever accessed school-based HIV education. In uMkhanyakude, 36% of men aged 15–34 years had ever had VMMC (*n* = 878; 33% of males aged 15–49 years, *n* = 1020) (data not shown).

### Uptake of multiple intervention categories

**Fig. 4:**
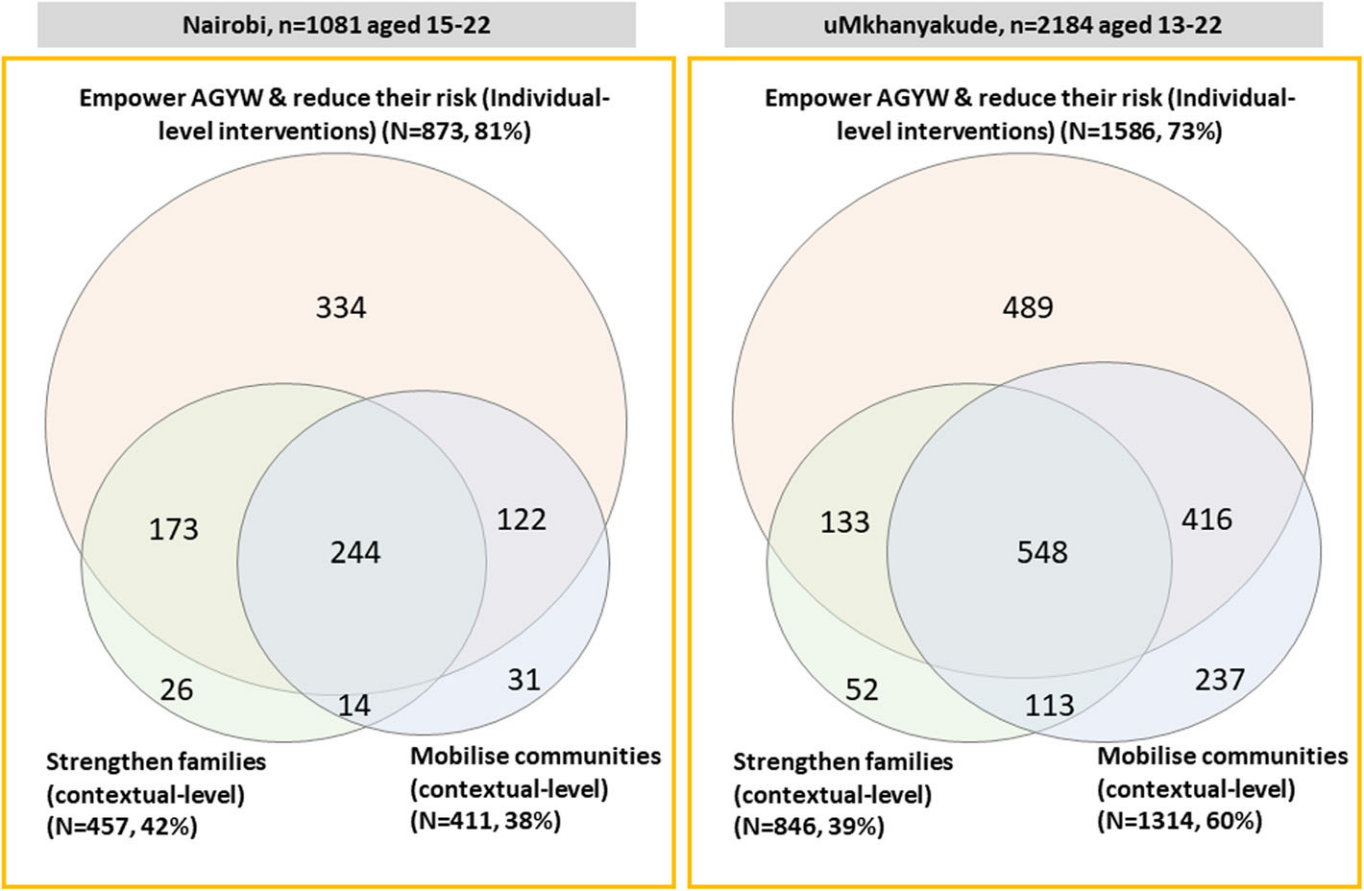
Layering of interventions across DREAMS core package levels in Nairobi and uMkhanyakude. Footnote: Numbers indicate those AGYW aged 15–22 in Nairobi and 13–22 in uMkhanyakude who used any intervention within each DREAMS core package intervention level in the last 12 months

The majority of AGYW had accessed interventions from multiple core package categories (Table 3), with > 60% accessing ≥2 categories and > 30% accessing ≥3 categories, in both younger and older AGYW and in both settings. AGYW aged 13–17 years accessed a greater number of categories compared to those aged 18–22 years. Over 50% of girls aged 10–14 years in Nairobi had accessed ≥2 categories and > 30% ≥3 categories (Table 2). Interventions were also frequently used in combinations across the individual and contextual levels, with > 60% of those aged 13–22 years using individual level interventions also participating in interventions that aim to strengthen the family or mobilise communities (Fig. 4).

**Table 3:** Invitation to DREAMS and uptake of core package, by characteristics and age-group, Nairobi and uMkhanyakude

Characteristics of AGYW	Nairobi, Kenya												uMkhanyakude, South Africa											
	Age 15–17 years						Age 18–22 years						Age 13–17 years						Age 18–22 years					
	Total	Invited	No. core package categories accessed				Total	Invited	No. core package categories accessed				Total	Invited	No. core package categories accessed				Total	Invited	No. core package categories accessed			
			0	1	2	3+			0	1	2	3+			0	1	2	3+			0	1	2	3+
	N	%	%	%	%	%	N	%	%	%	%	%	N	%	%	%	%	%	N	%	%	%	%	%
Overall (total)	547	58.9	14.8	19.0	18.1	48.1	534	40.1	10.5	24.0	25.7	39.9	1148	40.3	7.1	23.3	23.4	46.2	1036	17.0	10.7	24.5	30.5	34.3
Currently in school																								
No	88	48.9	19.3	23.9	18.2	38.6	367	33.2	10.1	25.9	30.8	33.2	20	30.0	10.0	45.0	30.0	15.0	520	11.5	13.5	31.9	34.2	20.4
Yes	459	60.8	13.9	18.1	18.1	49.9	167	55.1	11.4	19.8	14.4	54.5	1128	40.5	7.1	22.9	23.3	46.7	516	22.5	7.9	17.1	26.7	48.3
Marital status																								
Never/ previously married	535	59.4	15.0	18.9	17.8	48.4	341	48.1	12.3	22.9	19.6	45.2												
Currently married	12	33.3	8.3	25.0	33.3	33.3	193	25.9	7.3	25.9	36.3	30.6												
Geographic area																								
Rural													727	45.3	6.6	22.6	24.1	46.8	661	19.7	9.4	22.7	30.9	37.1
Peri-urban													351	34.8	7.4	22.2	21.7	48.7	309	12.0	12.9	27.5	30.4	29.1
Urban													60	10.0	11.7	38.3	23.3	26.7	57	14.0	14.0	24.6	31.6	29.8
Employment^a^																								
Yes	20	35.0	20.0	30.0	15.0	35.0	113	30.9	6.2	26.5	30.1	37.2	16	20.5	37.5	18.8	12.5	31.3	40	15.0	22.5	20.0	35.0	22.5
No	527	59.8	14.6	18.6	18.2	48.6	421	42.8	11.6	23.3	24.5	40.6	1129	40.6	6.6	23.4	23.6	46.4	990	17.2	10.2	24.6	30.4	34.7
Self-assessed household poverty																								
Very poor	66	66.7	6.1	9.1	21.2	63.6	73	45.2	9.6	16.4	19.2	54.8												
Moderately poor	435	58.4	16.3	20.7	17.0	46.0	423	40.2	10.2	24.6	26.5	38.8												
Not poor	46	52.2	13.0	17.4	23.9	45.7	38	28.9	15.8	31.6	28.9	23.7												
Received government grant																								
No													338	18.0	13.0	29.3	23.7	34.0	775	19.4	12.0	25.5	27.4	35.1
Yes (child-care/foster-child)													773	49.8	4.5	20.4	23.8	51.2	259	10.0	6.6	21.6	40.2	31.7
Socio economic status																								
Low													380	48.4	8.2	21.1	23.2	47.6	347	19.0	11.2	21.3	31.1	36.6
Middle													386	38.3	5.7	21.5	23.3	49.5	361	16.9	7.2	24.9	32.7	35.2
High													344	33.7	7.3	27.6	23.8	41.3	256	15.6	14.8	27.0	28.9	29.3
Food insecure^b^																								
No	351	55.3	15.4	19.9	18.2	46.4	366	33.6	11.2	26.8	28.1	33.9	898	41.1	6.7	24.1	23.2	46.1	603	21.2	11.3	26.2	27.9	34.7
Yes	185	67.0	13.5	15.7	17.8	53.0	165	54.5	8.5	17.6	20.6	53.3	250	37.6	8.8	20.4	24.4	46.4	432	11.1	9.7	22.2	34.3	33.8
Ever had sex																								
No	479	60.5	16.3	18.8	18.4	46.6	163	53.4	18.4	22.1	15.3	44.2	999	40.6	7.6	25.1	23.0	44.2	279	22.9	21.1	28.0	25.1	25.8
Yes	66	45.5	4.5	21.2	16.7	57.6	371	34.2	7.0	24.8	30.2	38.0	137	39.4	3.6	9.5	27.7	59.1	724	14.8	6.8	22.8	33.0	37.4
Ever pregnant																								
No	514	60.1	15.4	19.1	17.7	47.9	266	46.6	15.4	23.7	18.0	42.9	1077	40.9	7.4	23.8	23.3	45.5	499	20.4	16.2	28.9	26.9	28.1
Yes	31	35.5	6.5	19.4	25.8	48.4	268	33.6	5.6	24.3	33.2	36.9	63	31.7	1.6	14.3	28.6	55.6	494	14.0	5.3	20.2	33.8	40.7
Ever given birth																								
No	519	59.7	15.2	19.1	17.7	48.0	286	45.5	14.7	25.5	18.9	40.9	1099	40.5	7.3	23.7	23.4	45.6	548	20.1	15.9	29.9	26.5	27.7
Yes	26	38.5	7.7	19.2	26.9	46.2	248	33.9	5.6	22.2	33.5	38.7	42	35.7	2.4	11.9	28.6	57.1	469	13.4	4.5	18.3	35.6	41.6
Gender based violence^c^																								
No	321	57.9	20.2	16.2	18.1	45.5	286	40.9	11.9	25.9	24.5	37.8	715	40.1	9.0	22.7	23.5	44.9	697	15.1	12.5	25.3	29.7	32.6
Yes	226	60.2	7.1	23.0	18.1	51.8	248	39.1	8.9	21.8	27.0	42.3	433	40.6	4.2	24.2	23.3	48.3	339	20.9	7.1	23.0	32.2	37.8

Denominators shown are all girls in each age-group and characteristic category, regardless of invitation to participate

^a^ Nairobi: Not recently employed in last month vs. employed within last month; uMkhanyakude: Not employed currently vs. full or part time employment

^b^ Nairobi: Girl or household member went to sleep at night hungry because there was not enough food in past 4 weeks; uMkhanyakude: Girl or household member ever cut the size of their meal or skipped meals because there was not enough money for food

^c^ Nairobi: reported any of the following by a man in the past 12 months: humiliated; threatened to hurt or harm; insulted; pushed, shook, threw something; slapped; twisted arm or pulled hair; punched; kicked, dragged or beaten; tried to choke or burn; threatened to attack; attacked; unwanted sexual advances; attempted unwanted sex; forced sexual intercourse; forced sex acts

**Fig. 5:**
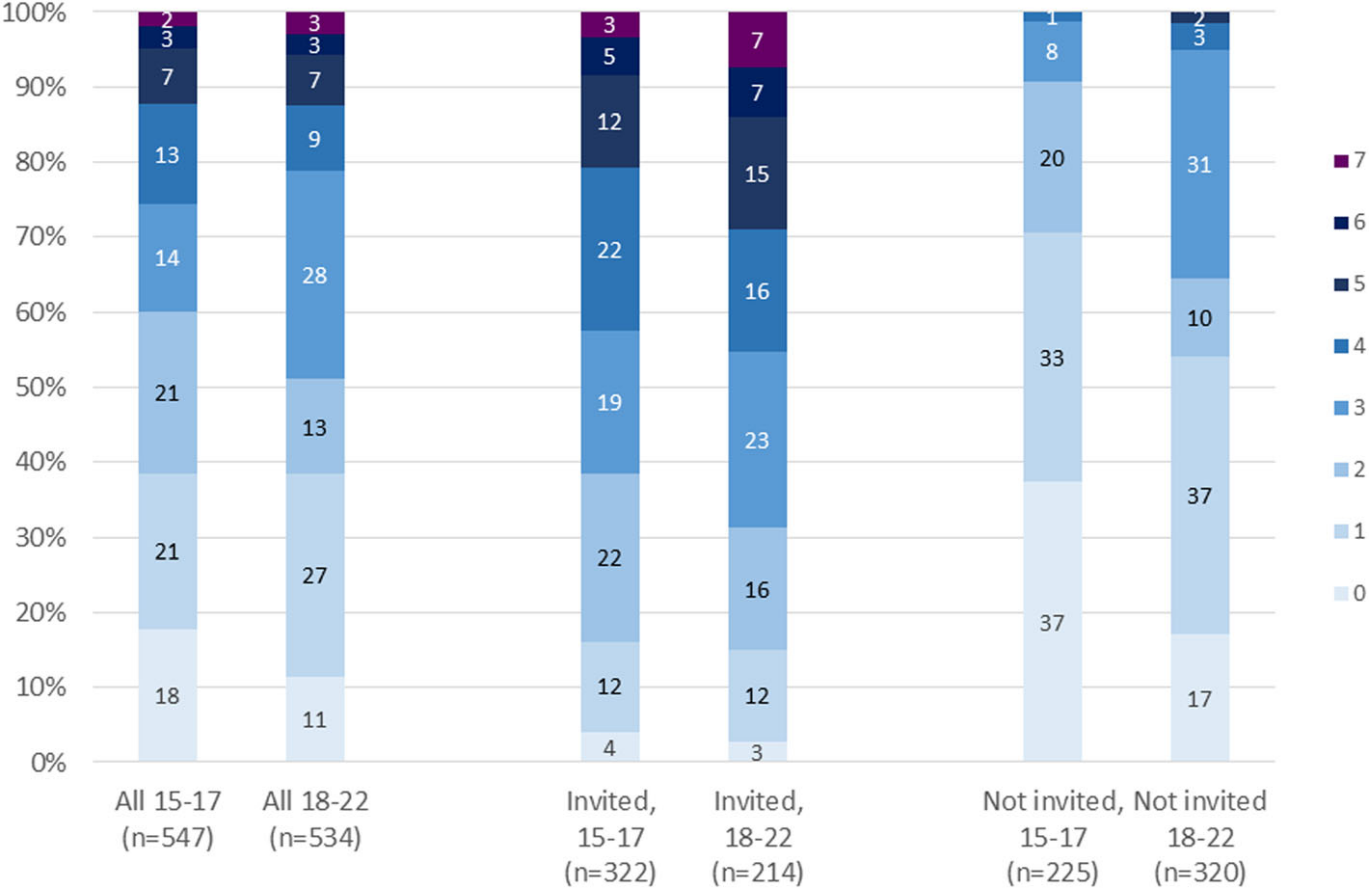
Number of primary interventions accessed, overall, and among those invited to DREAMS, by age, Nairobi. Footnote: Primary interventions in Kenya: HIV Testing Services, HIV and violence prevention, contraceptive method mix education, condom education and demonstration, financial capability training, entrepreneurship training, social asset building (PrEP excluded from the analysis - not asked on the 2017 Nairobi enrolment survey)

**Fig. 6:**
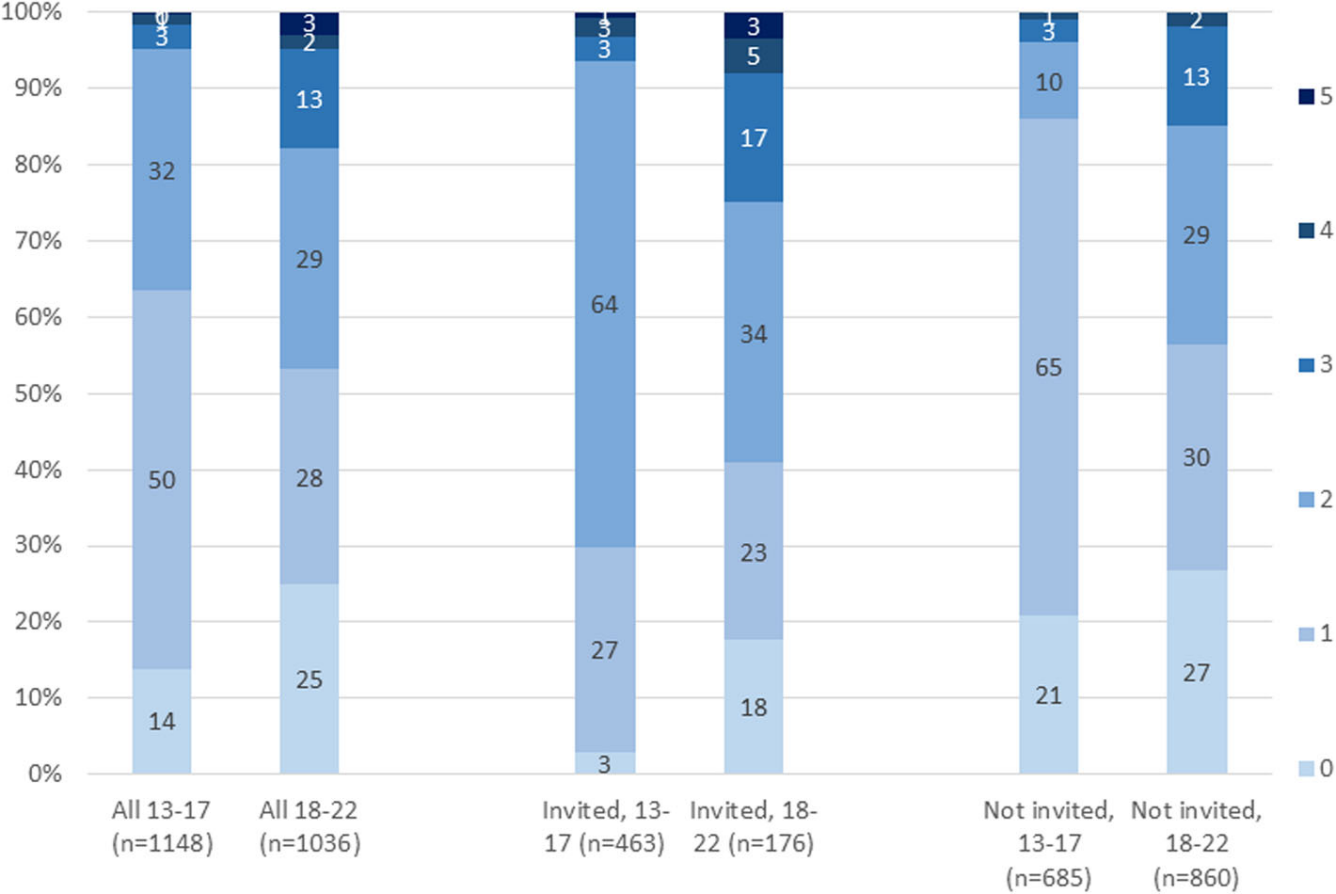
Number of primary interventions accessed, overall, and among those invited to DREAMS, by age, uMkhanyakude. Footnote: Primary interventions in South Africa: School-based HIV & violence prevention, social assets building (applicable to Non-sexually active and sexually active aged 10–19 years), Condoms, HIV testing and Sexual & reproductive health (applicable to sexually active only)

In terms of the ‘primary interventions’ specified by countries, the majority of AGYW in Nairobi had accessed at least two of them, although few had accessed all seven (Fig. 5). Findings were broadly similar for uMkhanyakude, where most AGYW had accessed at least two, but few had accessed all five intended primary interventions (Fig. 6).

### Uptake by characteristics of AGYW

Based on univariable analyses (Tables 3 and 4), self-reported invitation to participate in DREAMS was highest among the younger adolescent girls (13–17 years) than young women (18–22 years) in both Nairobi and uMhanyakude. AGYW were also more likely to be invited if they were: in school, had never had sex and were never pregnant. Higher proportions of AGYW who were not recently employed, never married, self-assessed as ‘very poor’, and food insecure were invited in Nairobi, as well as rural residents, recipients of government grants (among AGYW aged 13–17 years), those with low socio-economic status, and those who had migrated in uMkhanyakude. In general, associations between individual characteristics and invitation to participate in DREAMS followed the same pattern among older and younger AGYW. Overall, patterns for the number of intervention categories accessed were broadly similar to the patterns for invitation to participate, although those who had sex, or a pregnancy/birth, generally accessed more categories.

**Table 4:** Uni/multi-variable analyses of characteristics associated with invited to participate in DREAMS, Nairobi and uMkhanyakude

Characteristics of AGYW	Nairobi						uMkhanyakude					
	cOR	95% CI	p (LRT)	aOR	95% CI	p (LRT)	cOR	95% CI	p (LRT)	aOR	95% CI	p (LRT)
Age^a^												
13/15–17	1						1			1		
18–22	0.5	0.4–0.6	< 0.001				0.3	0.3–0.4	< 0.001	0.5	0.4–0.6	< 0.001
Marital status												
Never/previously married	1			1								
Currently married	0.3	0.2–0.4	< 0.001	0.4	0.3–0.7	< 0.001						
Urban or rural												
Rural							1			1		
Peri-urban/urban							0.6	0.5–0.7	< 0.001	0.6	0.5–0.7	< 0.001
Currently in school												
No	1			1			1			1		
Yes	2.6	2.0–3.3	< 0.001	1.7	1.3–2.4	< 0.001	3.8	2.9–5.1	< 0.001	1.9	1.4–2.7	< 0.001
Employment												
No	1			1								
Yes (in last month)	0.4	0.3–0.6	< 0.001	0.5	0.4–0.8	0.003						
Household poverty												
Very poor	1		0.05 for trend									
Moderately poor	0.8	0.6–1.1										
Not poor	0.6	0.3–1.0										
Socio-economic status												
Low							1			1		
Middle							0.7	0.6–0.9		0.7	0.6–0.9	
High							0.7	0.5–0.9	< 0.001	0.6	0.5–0.8	0.001
Food insecure^b^												
No	1			1			1			1		
Yes	2.0	1.5–2.6	< 0.001	2.0	1.5–2.7	< 0.001	0.5	0.4–0.7	< 0.001	0.6	0.5–0.8	< 0.001
Ever had sex												
No	1						1					
Yes	0.4	0.3–0.5	< 0.001				0.4	0.3–0.5	< 0.001			
Ever pregnant												
No	1						1			1		
Yes	0.4	0.3–0.5	< 0.001				0.4	0.3–0.5	< 0.001	0.8	0.6–1.0	0.07
Sexually exploited^c^												
No	1		0.6									
Yes	0.9	0.7–1.3										
Transactional sex												
No	1											
Yes	0.7	0.3–1.5	0.3									
Experienced violence^d^												
No	1						1					
Yes	1.0	0.8–1.2	0.8				1.2	1.0–1.5	0.04			
Migrated since age 13												
No							1					
Yes							0.5	0.4–0.6	< 0.001			

Also investigated: Nairobi: migration (lived elsewhere for > 1 month in last 3 months), ever given birth, has at least 1 child, number of individual assets, number of household assets, 2+ classes behind at school

Also investigated: uMkhanyakude: ever drank alcohol, cell phone use

*cOR* Crude odds ratio, *aOR* Adjusted odds ratio, *LRT* Likelihood ratio test

^a^ Nairobi: 15–17 and 18–22 years; uMkhanyakude 13–17 and 18–22 years

^b^ Nairobi: Girl or household member went to sleep at night hungry because there was not enough food in past 4 weeks; uMkhanyakude: Ever skipped or reduced a meal

^c^ either reported someone made them have sex against their will in the last 12 months, or reported any of the following by a male in the last 12 months: touched in a sexual way when unwanted; tried to have sex with you but did not succeed; forced you to have sex when you did not want to; forced you to perform sexual acts when you did not want to

^d^ Nairobi: reported any of the following by a man in the past 12 months: humiliated; threatened to hurt or harm; insulted; pushed, shook, threw something; slapped; twisted arm or pulled hair; punched; kicked, dragged or beaten; tried to choke or burn; threatened to attack; attacked; unwanted sexual advances; attempted unwanted sex; forced sexual intercourse; forced sex acts. uMkhanyakude: Ever experienced any form of violence

In Nairobi, higher proportions of AGYW aged 13–14 years were invited to and participated in DREAMS interventions compared to those aged 10–12 years (Table 2), and compared to those aged 18–22 years. AGYW aged 10–14 reporting socio-economic vulnerabilities (family often had insufficient food, or higher density sleeping arrangements) were more frequently invited into DREAMS, and participated in a greater number of intervention categories, compared with those not reporting such vulnerabilities.

In multivariable analyses, in both settings there was strong evidence for associations of schooling and food insecurity with invitation to participate in DREAMS (*p* < 0.001 for each), although those reporting hunger/reduced meals were *less* likely to be invited in uMkhanyakude, and *more* likely to be invited in Nairobi (Table 4; *p* < 0.001 for both). Age group was strongly associated with invitation to participate in uMkhanyakude (*p* < 0.001), with the older group (18–22 years) less likely to be invited, but not so in Nairobi after accounting for other characteristics. In Nairobi, never/previously married women (*p* < 0.001) and those not recently employed (*p* = 0.003) were more likely to be invited, as were those with rural residence (*p* < 0.001), low SES (*p* = 0.001) and ever pregnant (*p* = 0.07) in uMkhanyakude.

In multivariable analysis there was evidence that the following were associated with participation in ≥3 core package intervention categories: being in school (*p* < 0.001, both settings) and ever being pregnant (*p* = 0.008 Nairobi, *p* < 0.001 uMkhanyakude); not being married (*p* = 0.01), household poverty (*p* = 0.002), food insecurity (*p* = 0.002) and experience of sexual exploitation (*p* = 0.001) in Nairobi; and younger age group (*p* = 0.001), lower SES (*p* = 0.02) and ever had sex (*p* = 0.006) in uMkhanyakude (Additional files 9 and 10). Characteristics associated with participation in ≥4 intervention categories were largely similar.

## Discussion

As possibly the most ambitious example of combination HIV prevention to date, we sought to evaluate the extent to which DREAMS reached AGYW and related target groups in large and representative samples in diverse settings. Our findings are among the first to demonstrate that it is possible to deliver combination HIV prevention interventions to AGYW, in real-world (non-trial) settings, addressing biological, behavioural (sexual) and social protection pathways.

We found high levels of awareness and uptake of DREAMS among AGYW after 1 year of implementation in Kenyan and South African, urban and rural, settings, with the highest levels in Nairobi (where DREAMS had been implemented longest). In Gem, lower awareness reflected the earlier phase of DREAMS implementation at the time these data were collected. In contrast, awareness and uptake was low among other population groups, e.g. adult women and young men targeted for ‘contextual’ interventions in the DREAMS package.

The majority of AGYW beneficiaries accessed multiple categories of the core package, typically 2–3, which often included both individual and contextual-level interventions. This evidence of ‘layering’ indicates that programmatic integration across sectors is feasible. Findings from other studies in similar settings have also indicated that it is feasible and acceptable, though challenging, to deliver combination HIV prevention packages to AGYW, though these packages have usually combined either “health service”, *or* social, *or* behavioural interventions, rather than all three together. Examples of such initiatives include delivery of a prevention package including universal HIV testing and treatment to young people within the context of the PopART trial in Zambia [31]; and combination of microfinance, gender/HIV training and community mobilisation for women through the IMAGE trial in South Africa [32]. The EMPOWER trial in Tanzania and South Africa also offers useful insights by demonstrating the feasibility and acceptability of combining a wider array of interventions for AGYW, e.g. integrating gender-based violence screening with HIV testing services, delivering PrEP alongside sexual and reproductive health interventions, counselling, community/partner mobilisation, and empowerment clubs [33]. Our analyses extend these findings to non-trial settings and to ‘layering’ a comprehensive combination of biological, behavioural, and social interventions to AGYW and their partners/families. They also complement findings emerging from parallel research conducted by the Population Council on the effectiveness of efforts to recruit vulnerable AGYW to DREAMS, in a range of different communities [34–37]. However, detailed findings on awareness and ‘layering’ of the DREAMS core package and primary interventions have not been reported and there are methodological differences compared to our evaluation. Our study design leverages long-standing population-based demographic surveillance platforms, allowing for robust comparison groups (those not reached by DREAMS) and for analyses of uptake of relevant DREAMS interventions in the community.

Although we found evidence of ‘layering’, the intention of the DREAMS Partnership is to ‘layer’ more interventions than we observed, e.g., between 3 and 7 interventions in the primary intervention packages defined by age group. More time may be required to achieve greater ‘layering’, given the primary interventions were specified in the second year of DREAMS implementation (circa July 2017), and because roll-out of interventions was typically staggered until the full package became available [20]. In particular, interventions that were new to an area, e.g., ‘safe spaces’ and social asset building, or social protection interventions, took longer to implement than pre-existing interventions that were expanded with DREAMS funding, e.g., HIV testing services and school-based HIV education. This is reflected in the relatively lower uptake and ‘layering’ of the new interventions, in comparison to more established services.

In both Nairobi and uMkhanyakude, uptake of individual and ‘layered’ interventions was highest among younger women (particularly those aged 13/15–17 years) compared to 18–22 years. This indicates that DREAMS offers a model for reaching adolescents early, prior to entering a high risk window (e.g., before age 15/16, when HIV incidence starts to rise rapidly among girls [1]), and before most young women access health services for the first time (often with their first pregnancy). This was reinforced in Nairobi with the high receipt of DREAMS interventions, including multiple interventions, among 10–14 year olds – a stage dubbed the ‘window of opportunity’ by UNICEF [38]. On the other hand, the program was relatively less effective at reaching young women entering the period of ‘peak’ HIV risk (typically early/mid-twenties in high-burden settings [1]), which may impede its impact on HIV incidence, particularly over a short timeframe.

DREAMS was effective at reaching AGYW with socio-economic vulnerabilities, in both Kenya and South Africa, although less so for AGYW who were out of school than those in school. Invitation to participate was also lower among AGYW who had ever had sex or ever been pregnant, i.e., those at potentially higher sexual risk, although ‘layering’ was more common among these AGYW (which may reflect engagement in pre-existing antenatal care and reproductive health services). Research conducted by Population Council in Zambia and Kenya has also found under-representation of out-of-school and sexually active AGYW among DREAMS beneficiaries and, more generally, defined the majority of an out-of-school AGYW sample in Kenya as ‘lower’ vulnerability [34, 39].

These findings show that, while feasible to deliver multiple interventions for ‘layered’ HIV prevention, among large proportions of the general population of AGYW, challenges remained for higher coverage and greater ‘layering’ of DREAMS, including increasing coverage among older, out-of-school AGYW, and community-based programmes to reach families and men, if DREAMS is to impact on HIV incidence. These findings reflect that achieving scale-up of such a complex intervention, as fully intended, takes time, and suggest that phased implementation can allow time for reprioritisation, where required [24]. Some interventions - like community-based norms interventions and parenting programmes - were newly introduced in these settings, without programme infrastructure to build from. They therefore required intensive training of implementing partners and took longer to roll-out and scale-up than services with a pre-existing infrastructure [24]. Related qualitative research in South Africa has also revealed that conflicts with home priorities and logistical issues such as transport have contributed to challenges with recruiting and retaining AGYW and caregivers into DREAMS parenting programmes (who ideally participate together, at least in some sessions, but are often unable to do so in practice) [40]. Future rounds of our evaluation research will continue to track uptake by intervention type and sub-group, to assess changes over time.

Strengths of the study include the large, representative samples which enable accurate, population-level estimates of DREAMS’ reach among AGYW as well as important related target groups, across diverse settings. Harmonised research tools, with questions on all interventions in the core package, allowed for detailed assessment of combinations of interventions and comparable summaries across settings. Still, measuring uptake of such a complex programme with so many components is challenging. We relied on self-reported information on invitation to participate in DREAMS, as a marker of who was intended to benefit from, as well as who actually accessed, the programme. This may have underestimated participation, if some AGYW did not know they had been ‘invited to DREAMS’, or that interventions were DREAMS-funded. Going forward, linkages with individual-level programme data may improve our classification of which AGYW were DREAMS beneficiaries. Differences in the data available in the 2016 Gem survey, compared to data collected in 2017 in Nairobi and uMkhanyakude, limited some of the comparisons that could be made. For example, invitation to participate in DREAMS was not captured explicitly in Gem and participation was measured as ‘ever participated’, compared to participation within the last 12 months in the other two settings (although DREAMS was introduced in 2016, so participation in DREAMS-specific interventions in Gem should have reflected participation within the prior 12 months only). Furthermore, the heterogeneity of DREAMS implementation across settings limited the comparability of some measures, though we strove to standardise using common frameworks like the core package and primary intervention packages, as defined by PEPFAR [6]. The STROBE reporting guidelines were also used to guide synthesis and standardise reporting of our findings across settings [30] (Additional file 2). This evaluation focussed on selected DREAMS sites (justified in Birdthistle et al. [17]) and our findings may not be generalisable to all other DREAMS intervention sites.

## Conclusions

This study contributes detailed evidence to a relatively sparse body of research on the feasibility of scaling-up combination HIV prevention in non-trial conditions. Such evidence is important for understanding how to bridge the ‘implementation gap’ [15, 16]. Our findings reveal that it is possible to deliver multiple interventions at scale, among target populations of AGYW, including socio-economically vulnerable individuals, in varied settings. However, we showed that maximising ‘layering’ with the full range of intended interventions takes time, especially when interventions are being delivered in an area, or to a population, for the first time. This is particularly true among key sub-groups such as older and out-of-school AGYW, while efforts to reach male partners and families with community-level programmes also need to be intensified. Specifically, lessons here can inform programming that aims to maximise the impact of HIV prevention among young women, especially in the context of current expansion of DREAMS, ‘DREAMS-like’ programmes, and other multi-sectoral programming. Moreover, we will continue to track uptake over time as two further years of data collection (2018–2019), combined with ongoing process evaluation, will offer longer-term lessons about scale-up and sustainability, as well as impact.

## Supplementary information

**Additional file 1.** Summary of primary intervention packages in each country setting, by age.

**Additional file 2.** STROBE checklist.

**Additional file 3.** Characteristics of men aged 15–49 and women aged 25–49 years in Nairobi, uMkhanyakude and Gem general population surveys.

**Additional file 4.** Sources of information about DREAMS, among AGYW who ever heard of DREAMS, in Nairobi and uMkhanyakude.

**Additional file 5.** Sources of information about DREAMS, among men aged 15–49 years and older women aged 25–49 years who ever heard of DREAMS, by age, in Nairobi.

**Additional file 6.** Awareness and usage of specific DREAMS interventions among AGYW aged 15–17 in Nairobi.

**Additional file 7.** Awareness and usage of specific DREAMS interventions among AGYW aged 18–22 in Nairobi.

**Additional file 8.** Uptake of categorised interventions of the DREAMS Core Package in Kenya (Nairobi and Gem), among men by age group (panel A), and among women aged 25–49 (panel B).

**Additional file 9.** Univariable and multivariable analyses of AGYW characteristics associated with participation in 3+ or 4+ DREAMS core package intervention categories in the last 12 months in Nairobi.

**Additional file 10.** Univariable and multivariable analyses of AGYW characteristics associated with participation in 3+ or 4+ DREAMS core package intervention categories in the last 12 months in uMkhanyakude.

## Data Availability

Data underlying published results will be accessible and open, subject to a transition period (available from the London School of Hygiene and Tropical Medicine data repository https://datacompass.lshtm.ac.uk by contacting researchdatamanagement@lshtm.ac.uk), as per the Open Access Policy of the Bill & Melinda Gates Foundation.

## Abbreviations

AGYW: Adolescent girls and young women
DREAMS: Determined, Resilient, Empowered, AIDS-free, Mentored, and Safe lives
NUHDSS: Nairobi Urban Health and Demographic Surveillance System
PEPFAR: President’s Emergency Fund for AIDS Relief
PrEP: Pre-exposure Prophylaxis
VMMC: Voluntary medical male circumcision
