# Supplementary material for: Awareness and uptake of layered HIV prevention programming for young women: analysis of population-based surveys in three DREAMS settings in Kenya and South Africa
Source: BMC Public Health. 2019 Oct 30;19:1417. doi: 10.1186/s12889-019-7766-1 (PMC6824290; doi:10.1186/s12889-019-7766-1)
Supplement: Supplementary file 1 — Additional file 1. Summary of primary intervention packages in each country setting, by age. [file 12889_2019_7766_MOESM1_ESM.docx]

**Additional file 1. Summary of ‘primary’ intervention packages in each country setting, by age**

| **Setting** | **List of constituent interventions in ‘primary’ intervention packages** |
| --- | --- |
| Kenya | - HIV Testing and Counselling (age 10-24) - Social asset building (age 10-24) - School-based HIV and violence prevention (age 10-24) - Financial capability training (age 10-24) - Entrepreneurship training (age 15-24) - Condom education and promotion (age 15-24) - Contraceptive method mix education and counselling (age 15-24) - PrEP education and counselling (age 18-24) |
| South Africa | - School-based HIV and violence prevention (age 10-19) - Social asset building (age 10-19) - Condoms (sexually active age 10-24) - HIV Testing and Counselling (sexually active age 10-24) - Sexual and reproductive health (sexually active age 10-24) |
