# Supplementary figures and images for: Awareness and uptake of layered HIV prevention programming for young women: analysis of population-based surveys in three DREAMS settings in Kenya and South Africa
Source: BMC Public Health. 2019 Oct 30;19:1417. doi: 10.1186/s12889-019-7766-1 (PMC6824290; doi:10.1186/s12889-019-7766-1)

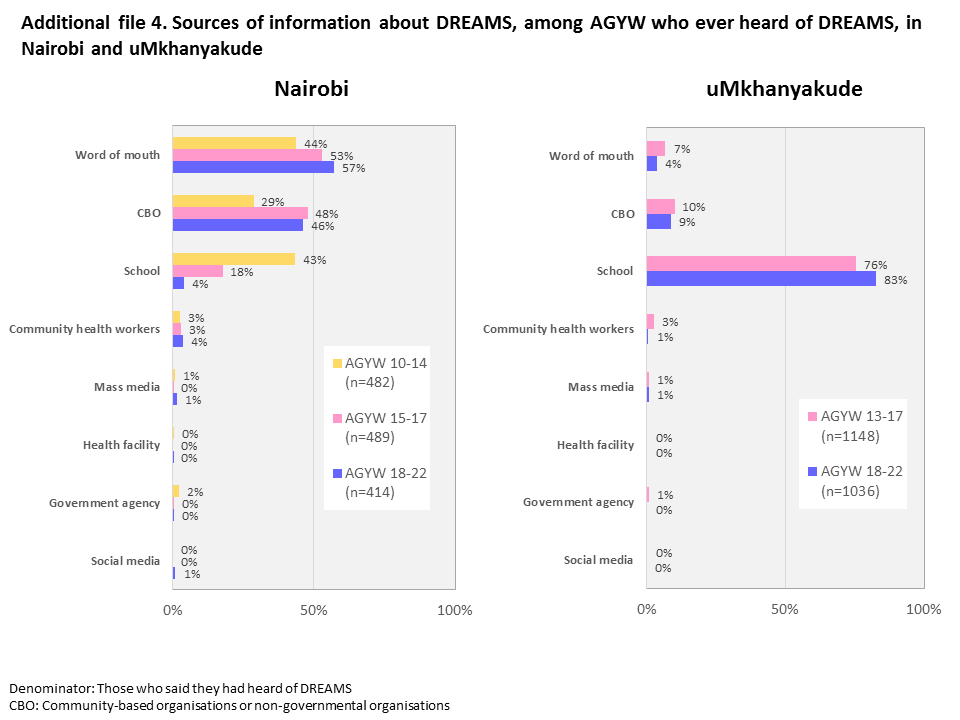

Supplement: Supplementary file 4 — Additional file 4. Sources of information about DREAMS, among AGYW who ever heard of DREAMS, in Nairobi and uMkhanyakude. [file 12889_2019_7766_MOESM4_ESM.tif]

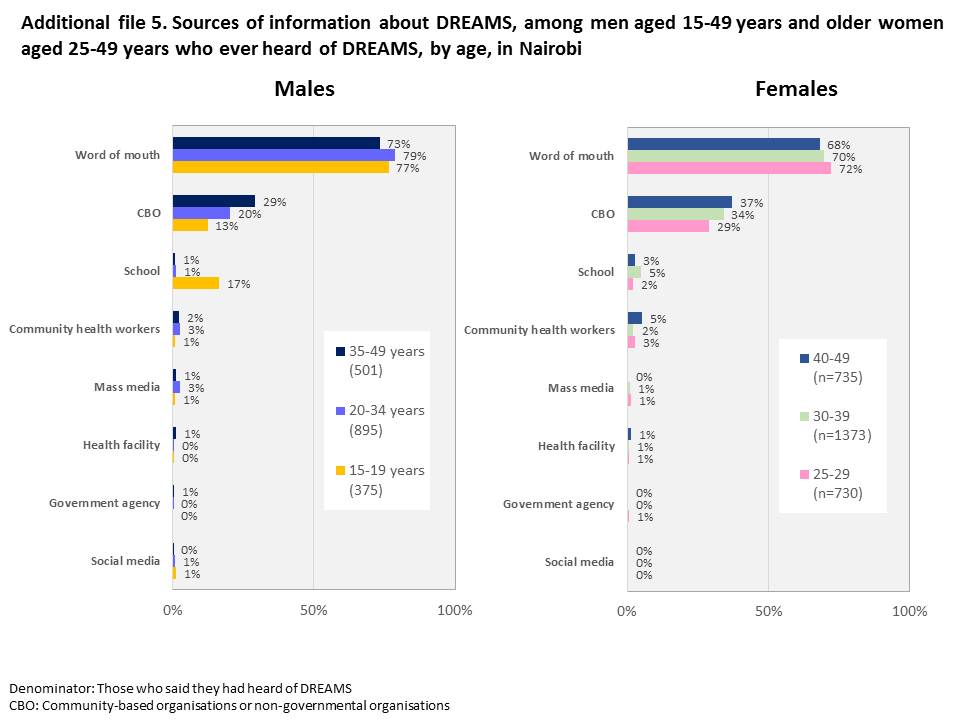

Supplement: Supplementary file 5 — Additional file 5. Sources of information about DREAMS, among men aged 15–49 years and older women aged 25–49 years who ever heard of DREAMS, by age, in Nairobi. [file 12889_2019_7766_MOESM5_ESM.tif]

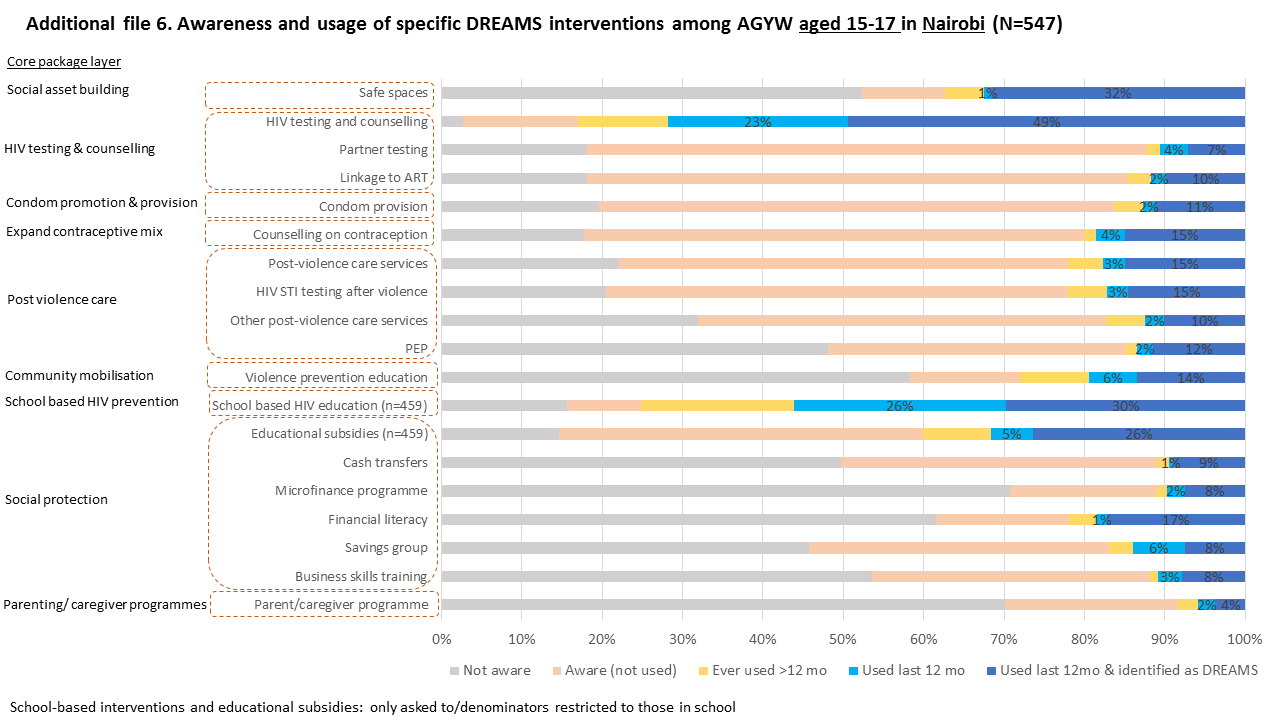

Supplement: Supplementary file 6 — Additional file 6. Awareness and usage of specific DREAMS interventions among AGYW aged 15–17 in Nairobi. [file 12889_2019_7766_MOESM6_ESM.tif]

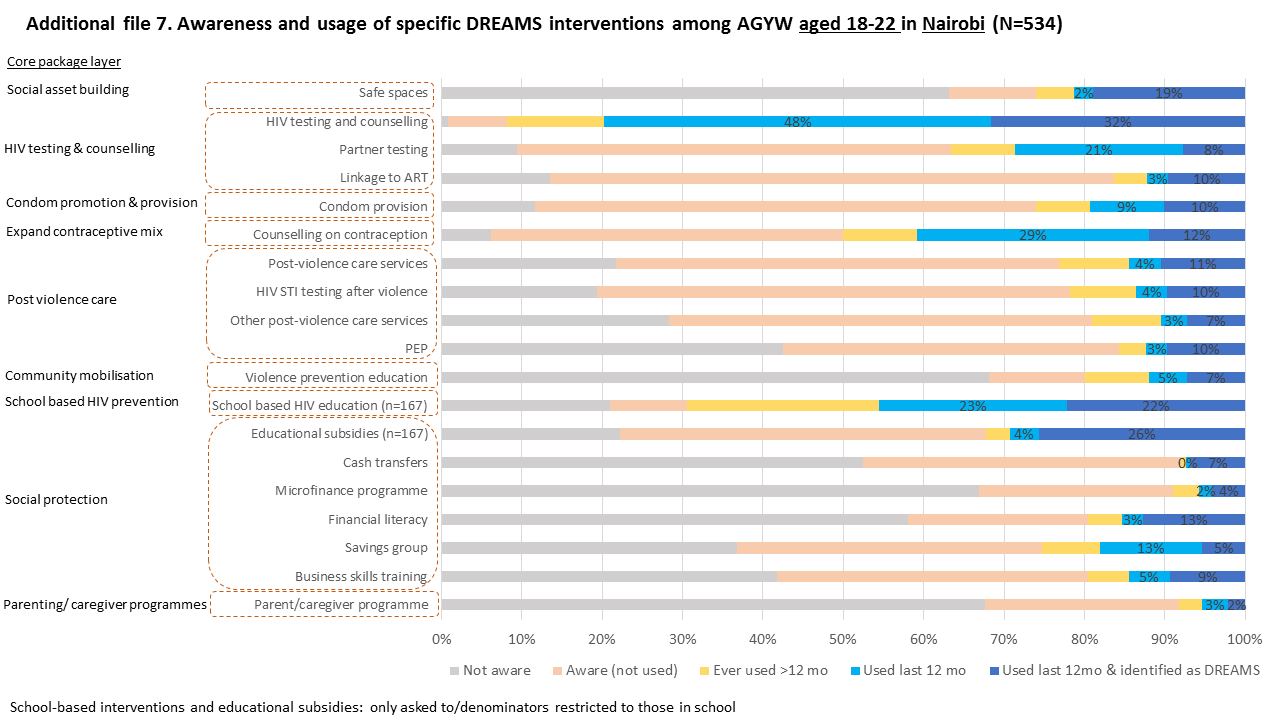

Supplement: Supplementary file 7 — Additional file 7. Awareness and usage of specific DREAMS interventions among AGYW aged 18–22 in Nairobi. [file 12889_2019_7766_MOESM7_ESM.tif]

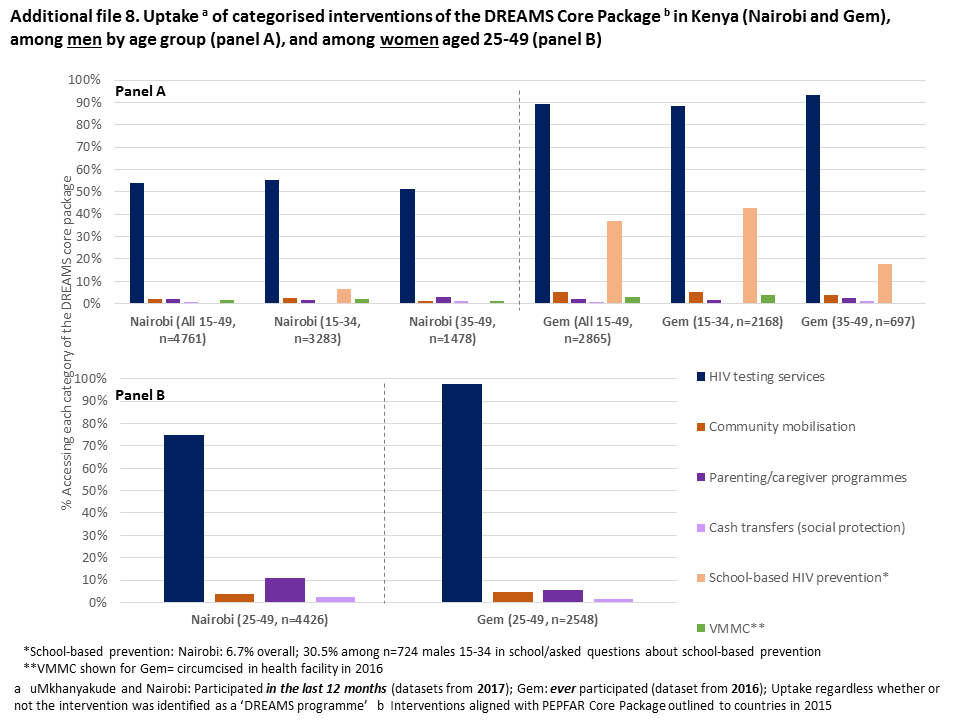

Supplement: Supplementary file 8 — Additional file 8. Uptake of categorised interventions of the DREAMS Core Package in Kenya (Nairobi and Gem), among men by age group (panel A), and among women aged 25–49 (panel B). [file 12889_2019_7766_MOESM8_ESM.tif]
